# The mediating effect of mindfulness on demoralization syndrome and quality of life of thyroid cancer patients: A correlational study: Erratum

**DOI:** 10.1097/MD.0000000000042558

**Published:** 2025-05-09

**Authors:** Li YuYu, Zhao Shan, Peng JingJun

In the article “The mediating effect of mindfulness on demoralization syndrome and quality of life of thyroid cancer patients: A correlational study”^1^, which appears in Volume 102, Issue 7 of *Medicine* the affiliation for all authors is incorrect. The correct affiliation is “Department of Thyroid and Hernia Surgery, Guangdong Provincial People’s Hospital (Guangdong Academy of Medical Sciences), Southern Medical University, Guangzhou, China.” The correspondence information is also incorrect. The correct affiliation information is: “Peng JingJun, Department of Thyroid and Hernia Surgery, Guangdong Provincial People’s Hospital, No. 106 Zhongshan Second Road, Yuexiu District, Guangzhou, Guangdong Province, 510080, China (e-mail: pengjingjun123@163.com)”.

## References

[1] YuYu L Shan Z JingJun P. The mediating effect of mindfulness on demoralization syndrome and quality of life of thyroid cancer patients: a correlational study. Medicine (Baltimore). 2023;102:e32719. 36800585 10.1097/MD.0000000000032719 PMC9936027

